# Fractional Flow Reserve Derived from a Single Angiographic View: Fact or Fiction?

**DOI:** 10.3390/medicina62030434

**Published:** 2026-02-25

**Authors:** Michail I. Papafaklis, Anastasios Papoutsoglou, George C. Bourantas, Grigorios Tsigkas, Konstantinos Katsanos, Antonios Karanasos, Foivos V. Bekiris, Periklis Davlouros

**Affiliations:** 1 Faculty of Medicine, School of Health Sciences, University of Patras, 26504 Rio, Greece; gregtsig@upatras.gr (G.T.); katsanos@med.upatras.gr (K.K.); akaranasos@upatras.gr (A.K.); pdav@upatras.gr (P.D.); 2 Cardiology Division, University Hospital of Patras, 26504 Rio, Greece; a.papoutsoglou@upatras.gr; 3 Medlytic Labs, 26222 Patras, Greece; georgios.bourantas@gmail.com (G.C.B.); fbekiris@gmail.com (F.V.B.); 4 Department of Interventional Radiology, University Hospital of Patras, 26504 Rio, Greece

**Keywords:** angiography, coronary artery disease, computation, catheterization, functional assessment

## Abstract

Accurate assessment of the functional significance of coronary artery stenoses is essential for guiding revascularization decisions and improving clinical outcomes in patients with coronary artery disease (CAD). While invasive wire-based fractional flow reserve (FFR) remains the gold standard for physiological lesion assessment, its adoption in routine clinical practice is limited by procedural complexity, patient discomfort, time consumption, and cost. These limitations have driven the development of angiography-derived FFR techniques that enable physiological evaluation without pressure wires or pharmacologic hyperaemia. Recent advances in computational modelling, artificial intelligence, and image processing have facilitated the estimation of FFR from conventional coronary angiography, including approaches that require only a single angiographic view. Single-view angiography-derived FFR methods—such as Murray law-based quantitative flow ratio (µQFR), FFR2D, Angio-iFR/FFR, sAccuFFR, and X1-FFR—aim to simplify workflow while maintaining diagnostic accuracy. Among these, µQFR has demonstrated the most consistent validation against invasive FFR across a broad range of clinical scenarios, including complex lesions, severe aortic stenosis, multivessel disease, and acute coronary syndromes. This review summarizes the principles, validation data, clinical applications, and limitations of single-view angiography-derived FFR technologies and highlights their potential to expand the adoption of physiology-guided coronary intervention.

## 1. Introduction

Coronary artery disease (CAD) remains a leading cause of global morbidity and mortality, underscoring the need for accurate assessment of coronary artery stenoses [1]. In patients with systemic cardiovascular risk factors, progressive atherosclerosis leads to the development of coronary lesions that may manifest as acute or chronic coronary syndromes. Identification of hemodynamically significant stenoses is critical, as it directly influences revascularization strategies and ultimately determines patient outcomes. Conventional coronary angiography provides detailed anatomical information but is limited in its ability to assess the functional significance of intermediate coronary lesions. The severity of lesions, either estimated visually or by quantitative coronary angiography (QCA), is a poor correlate of actual blood flow limitation, especially in the setting of intermediate-grade stenoses. This problem can lead to deferring revascularization of functionally relevant lesions that require intervention or undertaking unnecessary procedures for anatomically intermediate lesions that do not limit myocardial blood flow, thereby subjecting patients to unwarranted risks and expenses [2]. Although non-invasive stress imaging was initially regarded as the reference standard for evaluating the functional significance of stenoses, diagnostic accuracy is limited in multivessel disease, and it cannot determine lesion-specific ischemia in complex coronary anatomy with multiple stenoses [3].

Fractional flow reserve (FFR), defined as the ratio of maximal achievable blood flow in a diseased coronary artery to the theoretical maximal flow in the absence of stenosis, is measured under hyperaemic conditions and has become the gold standard for assessing the functional significance of coronary lesions [4]. The fundamental objective of FFR-based assessment is to discriminate ischemia-producing from non-ischemia-producing lesions, ensuring that revascularization is directed toward patients most likely to benefit. Numerous clinical trials have demonstrated that FFR-guided percutaneous coronary intervention (PCI) improves clinical outcomes and cost-effectiveness compared with angiography-guided PCI, a benefit that has been incorporated into contemporary clinical guidelines [5,6].

Despite the robust evidence base, conventional wire-based FFR has not been universally adopted in routine practice. Large registry data show only a modest increase in its utilization over the past decade, with substantial variability across regions and catheterization laboratories [7]. The reasons for limited uptake include the invasive nature and risk of pressure-wire manipulation [8,9], the need for hyperaemic agents with associated patient discomfort and potential adverse effects [4,10,11], increased procedural time and radiation exposure [3,8,12], additional costs [4], and the requirement for specialized training [13]. Although the requirement of pharmacological agents for inducing hyperaemia has been addressed during the last decade by the introduction of the instantaneous wave-free ratio (iFR) [6], all other limitations remain. These barriers have stimulated intensive research into less invasive alternatives capable of providing reliable physiological assessment without compromising diagnostic accuracy.

Angiography-derived FFR methodologies have emerged as a paradigm shift in interventional cardiology during the last 15 years [14]; by leveraging advances in imaging, computational fluid dynamics (CFD), and artificial intelligence (AI), these methods estimate FFR directly from conventional coronary angiograms (i.e., functional angiography) without the need for pressure wires or pharmacologically induced hyperaemia [1,2,3,4,8,12,14]. The central concept involves three-dimensional reconstruction (3D) of coronary anatomy from two or three angiographic images, followed by application of fluid mechanics principles to estimate pressure gradients across stenoses. Several studies have validated this approach compared to the gold standard (i.e., FFR) and have also shown benefits in clinical outcomes compared to traditional angiographic guidance [15,16]. In an effort to further simplify and streamline workflow in the catheterization laboratory, the latest advancement in angiography-based FFR approaches involves the use of a single angiographic view for functional assessment of coronary stenoses [8,17].

**Figure 1:**
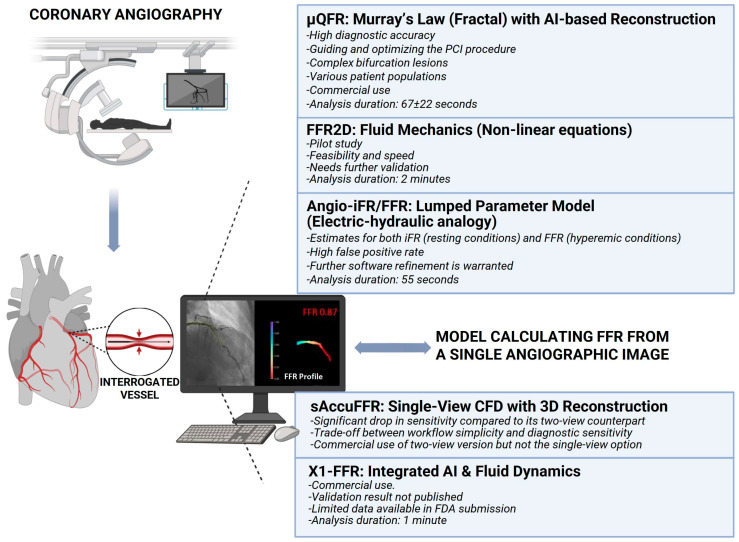
Overview of single-view angiography-derived fractional flow reserve (FFR) methodologies. Information on validation data and results, as well as analysis duration and major limitations are noted.

The purpose of this review is to provide a comprehensive overview and critical appraisal of single-view angiography-derived FFR methodologies (Figure 1 and Table 1). We critically evaluate their diagnostic accuracy compared with invasive reference standards and their computational counterparts from two/three angiographic images, and discuss current and future clinical applications. By synthesizing contemporary evidence, this review highlights the evolving role of single-view-based functional assessment in modern interventional cardiology.

## 2. Single-View Angiography-Derived FFR Approaches

### 2.1. Murray Law-Based Quantitative Flow Ratio (μQFR)

The original quantitative flow ratio (QFR) requires the acquisition of two separate optimal angiographic views, which is not always feasible and may increase radiation exposure. Murray law-based QFR (µQFR) represents a significant advancement by enabling accurate estimation of FFR from a single angiographic view [19,20]. This methodology is built upon the physiological principle of Murray’s law, which states that for optimal energy efficiency in a vascular tree, the cube of the radius of a parent vessel equals the sum of the cubes of the radius of its daughter branches [20,21].

The μQFR workflow integrates AI-driven image processing with established physiological principles [21]. From a single high-quality angiographic projection, the algorithm automatically delineates the lumen contours of the target vessel and its side branches, reducing operator dependency and improving reproducibility. Contrast flow velocity is derived from frame count analysis and used to estimate baseline coronary flow. The resting coronary flow is used to estimate the flow under hyperaemia, which is essential for calculating a pressure-drop equivalent to FFR. Importantly, instead of assuming a simple linear taper, the software reconstructs the “healthy” reference vessel diameter using Murray’s bifurcation fractal law. This law describes the natural, non-linear “step-down” in diameter that occurs as a main vessel divides into side branches. This provides a more physiologically accurate model, especially for lesions located at or near bifurcations. Pressure loss across the stenosis is calculated by applying basic fluid mechanics equations, which incorporate both viscous friction and inertial losses related to abrupt flow expansion, yielding the final μQFR value.

In a post hoc analysis of the FAVOR II China study, Tu et al. evaluated μQFR against invasive wire-based FFR in 306 patients (330 vessels) and demonstrated excellent correlation and agreement [21]. For identifying functionally significant stenoses (FFR ≤ 0.80), μQFR achieved a diagnostic accuracy of 93.0% (sensitivity: 87.5% and specificity: 96.2%). Intra- and interobserver variability was minimal (0.00 ± 0.03), underscoring the robustness of the automated approach. By requiring only a single view, μQFR overcomes the primary limitation of systems that require more than one view, which can fail in up to 18% of cases due to the inability to acquire a second view of good quality or errors/failure of 3D reconstruction of coronary anatomy [22]. The μQFR methodology appears particularly well suited for complex anatomy, including bifurcation lesions, because of the use of Murray’s law and the ability to simultaneously assess both the main vessel and its side branches. However, we must note that the study by Tu et al. did not include comparative invasive FFR measurements of side branches to provide full validation data [21].

The transition from two angiographic images to a single one raises the question of equivalence between the two approaches. Cortes et al. compared μQFR with 3D-QFR and showed absolute dichotomous agreement, i.e., there was 100% agreement between the two methods for classifying lesions as hemodynamically significant (≤0.80) or not; there were no false positives or false negatives [20]. In a post hoc analysis of the FAVOR II China study, 3D-μQFR (based on 3D reconstruction) as well as μQFR1 and μQFR2 (based separately on the first and second angiographic images) were successfully computed in 280 vessels from 262 patients to identify hemodynamically significant stenoses. Diagnostic accuracy for μQFR1 was 92.1%, for μQFR2 was 92.5%, and for 3D-μQFR was 92.5% [23]. With almost identical absolute values and diagnostic accuracy between the 2D- and 3D-derived computations, there may not be much to be gained from 3D-vessel reconstruction based on two angiographic views.

The single-view μQFR has also been evaluated in challenging clinical settings. In patients with severe aortic stenosis and concomitant coronary artery disease, invasive FFR may be unreliable since the high left ventricular pressures and altered hemodynamics can mask the true severity of coronary lesions, leading to false negative values. Yuta et al. demonstrated that μQFR before transcatheter aortic valve replacement (TAVR) correlated significantly with post-TAVR invasive FFR (r = 0.73), achieving an overall diagnostic accuracy of 84.2% with high specificity (96%) but modest sensitivity (62%) [9]. However, interpretation of these results requires caution owing to the retrospective study design with a very small sample size of 25 patients and the early timing of post-TAVR reference FFR measurements. FFR values may be even lower during the more chronic phase following TAVR and might underestimate the true rate of ischemia, potentially affecting the reported accuracy of μQFR [24]. Lastly, pre-TAVR angiograms were performed without nitroglycerin, which is generally recommended for μQFR analysis and may have influenced the results [9]. Nonetheless, μQFR may offer a promising alternative in this complex patient population.

Additional studies have explored μQFR in patients with intermediate coronary stenoses who undergo valvular heart surgery; the decision to perform concomitant coronary artery bypass grafting (CABG) in this patient subset is often debated. In a retrospective study with 486 patients undergoing valvular surgery with concomitant intermediate coronary lesions, functional incomplete revascularization (defined as leaving functionally significant lesions untreated based on μQFR) was an independent predictor of adverse outcomes, with these patients facing a higher risk of major adverse cardiac events [25].

The use of μQFR in complex coronary artery disease shows a consistent diagnostic performance across different types of complex lesions (bifurcation lesions: area-under-the-curve [AUC] of 0.95; tandem lesions: AUC of 1.00; calcified lesions: AUC of 0.97) [26]. Coronary calcification has been a major limitation for angiography-derived physiological assessments, because calcium deposits can interfere with contour detection. The performance of μQFR in this subset was assessed in the DIAMOND Study (Diagnostic Accuracy of Angiography-Derived Murray Law-Based Quantitative Flow Ratio in Moderate to Severe Calcified Coronary Lesions) showing moderate diagnostic accuracy (sensitivity and specificity of 75.0% and 77.8%, respectively) in a cohort of 107 patients and 120 vessels with angiographically confirmed moderate to severe calcification (ACC/AHA type B2 or C lesions) [27]. Although these values are lower than those reported in non-calcified vessels, the study indicates that μQFR remains a practical and useful tool even in complex calcified anatomy, offering a non-invasive alternative when wire manipulation is technically difficult or hazardous.

The management of non-culprit lesions in patients presenting with ST-segment myocardial infarction (STEMI) and multivessel disease is of particular interest in clinical practice and several randomized controlled trials. Invasive FFR assessment in non-culprit vessels in patients with STEMI in the acute phase can be unreliable due to microvascular dysfunction. Li, et al. investigated whether μQFR has a role in this setting; there was a good linear correlation between acute (i.e., calculated from the angiogram taken during the primary PCI) and staged (i.e., calculated from a follow-up angiogram during staged PCI before hospital discharge) μQFR, with a sensitivity of 87.7% and a specificity of 90.8% for detecting significant lesions in the acute phase [10].

Beyond diagnostic performance, μQFR has been investigated as a tool for PCI planning and optimization. In the QUITE RIGHT (Quantitative Flow Ratio Virtual Stenting and Angiography Guided Percutaneous Coronary Intervention) prospective randomized controlled trial, a strategy including planning based on μQFR and virtual PCI (allowing the operator to virtually implant stents of different lengths and diameters) for predicting the post-PCI μQFR before the actual procedure was compared directly against the conventional strategy of relying on the operator’s visual assessment of the angiogram for selecting a stent. A post-PCI μQFR ≥ 0.90 was achieved in 93.7% of the μQFR-guided pre-planning group versus 84.9% in the group with the conventional strategy. The μQFR-guided group also had a significantly lower contrast agent dose, a lower radiation dose, and a more appropriate stent length. At a median follow-up of 15.5 months, there was no statistically significant difference in major adverse cardiovascular events between the two groups. However, the study was powered for the physiological endpoint, not for clinical outcomes, and thus, longer follow-up is needed [28].

A post-hoc analysis of 806 vessels (777 patients) from the FLAVOUR (Fractional Flow Reserve and Intravascular Ultrasound-Guided Intervention Strategy for Clinical Outcomes in Patients with Intermediate Stenosis) trial further demonstrated that suboptimal post-PCI μQFR values (<0.90) independently predicted higher rates of target vessel failure at two years (6.1% compared to 2.7% in vessels with optimal results), highlighting its potential prognostic utility post-procedure [29]. Of note, while pre-procedure agreement with invasive FFR was very good, the diagnostic concordance between post-PCI μQFR and post-PCI wire-based FFR (available in a subset of only 262 vessels) was mediocre at 61%. Despite this difference, the post-PCI μQFR value itself remained a strong independent predictor of long-term clinical outcome.

Although µQFR has proven its diagnostic performance and clinical utility, there are a few limitations that should be taken into consideration. Anatomical complexity remains a challenge; diagnostic sensitivity is relatively reduced in moderate to severe calcified lesions. Similarly, while pre-PCI agreement with invasive FFR is high, post-PCI concordance with wire-based FFR—as seen in a post hoc analysis of the FLAVOUR trial—was moderate at 61%. This suggests that wire-based assessment might still be necessary for immediate post-stenting optimization in certain cases. Importantly, much of the supporting evidence in patient subsets relies on retrospective data and small-medium sample sizes; thus, larger prospective studies are needed to establish the role of µQFR across all clinical scenarios.

### 2.2. FFR2D

FFR2D represents a single-view approach that derives FFR directly from a conventional two-dimensional angiogram without 3D reconstruction or CFD simulation [30]. The method is built on established non-linear mathematical equations describing pressure-flow relationships in stenosed arteries. The process begins with the standard 2D coronary angiogram of the artery of interest, taken at an optimal viewing angle with as minimal vessel overlap or foreshortening as possible. The patient’s mean arterial pressure (Pa) is also recorded. Coronary artery contours are semi-automatically delineated, and key geometric parameters of the vessel, i.e., the reference vessel diameter and the minimum lumen diameter at stenosis, are obtained. Resting-state velocity flow rate is estimated using the TIMI frame count technique. Hyperaemic conditions are inferred mathematically rather than pharmacologically induced based on regression models derived from experimental data. Using the geometric information obtained from segmentation and the flow rates, including resting and simulated hyperaemic conditions, FFR2D applies fluid dynamics equations based on the work of Gould and Young et al. to calculate the pressure drop (ΔP) through the stenosis [31,32,33]. The equations take into account energy dissipation due to viscous friction, turbulence of flow, and flow separation. Then, the FFR2D is determined by applying the standard FFR formula: FFR2D = Pd/Pa, where Pd (distal pressure) is obtained by deducting the calculated pressure drop (Δp) from the initial mean arterial pressure (Pa). The total time required for this final calculation is noted to be merely 0.1 s [30].

In the pilot study by Tsigkas et al. [30], FFR2D demonstrated very high feasibility (96.7%) with very low exclusion rate; there was also good diagnostic accuracy compared with invasive FFR, correctly classifying 90.9% of lesions in 88 patients. The computed FFR2D values showed a good correlation with invasive FFR (r = 0.68, *p* < 0.001) and great concordance on a Bland–Altman plot with a very small mean difference of 0.000 ± 0.048. Using a cut-off value of ≤0.80, FFR2D achieved a sensitivity of 85.7% and specificity of 93.3%.

It is worthwhile noting a few issues regarding the FFR2D method. Firstly, in the pilot study and preliminary version of the software, the segmentation of the vessel is semi-automatic and requires user interaction for contour detection and correction; TIMI Frame Count is also manually performed by the operator. These can lead to variability between different operators and therefore influence the reproducibility of the results. The FFR2D pilot study mostly included LAD lesions (very few cases of the right coronary or left circumflex arteries), and the results were derived from a small, single-centre, retrospective dataset. Lastly, the study excluded patients with ostial or diffuse lesions, severe tortuosity, and poor image quality, potentially limiting its applicability in real-world practice. Otherwise, FFR2D could prove to be a valuable tool in the catheterization laboratories for fast, on-site physiology guidance.

The results of FFR2D are promising, but there are some technical and clinical limitations. The current semi-automatic vessel segmentation may lead to interobserver variability, which may affect reproducibility. The pilot study was a small, single-center retrospective study, predominantly involving LAD lesions, and the accuracy of the method for other coronary artery territories is not well demonstrated. The exclusion of more complex anatomical lesions indicates the need for further development.

### 2.3. Angio-iFR/FFR

The Angio-iFR/FFR system (Royal Philips, Amsterdam, the Netherlands) represents another methodological approach for coronary physiology assessment from a single angiographic view using a simplified, computationally efficient model [12]. The REVEAL iFR trial was a prospective, multicenter, international validation study designed to rigorously assess the diagnostic accuracy of this novel software against the gold standards of wire-based instantaneous wave-free ratio (iFR) and FFR. The Angio-iFR/FFR system uses a lumped parameter fluid dynamics model, which simplifies the complex coronary system by employing an “electric-hydraulic” analogy. The software models the coronary artery as an electrical circuit, where blood flow is analogous to current, pressure to voltage, and vascular resistance to electrical resistance. The resistance of each vessel segment is calculated using established fluid dynamics principles, including Poiseuille’s Law, Darcy–Weisbach friction, and Borda–Carnot expansion loss variables in order to calculate pressure losses from friction and expansion at stenoses. All anatomical data are derived from a single angiographic view, which allows for extremely rapid computation. A unique feature is the ability of the system to provide estimates for both iFR (resting conditions) and FFR (hyperemic conditions) using two separate computational models [12,33].

The REVEAL iFR trial enrolled 441 patients in 32 centres in Europe, Japan, and the United States and successfully analysed 97% of enrolled patients. Despite the theoretical advantages, the trial’s primary diagnostic performance goals were not met. Angio-iFR met a sensitivity of 77% with a notably poor specificity of 49%, falling significantly short of the 80% target with a high rate of false positives where non-significant lesions were incorrectly identified as significant. Similarly, Angio-FFR met a sensitivity of 78% and a specificity of 48%. The weak diagnostic performance was further confirmed by a poor correlation between Angio-iFR and wire-based iFR and a low area-under-the-curve of 0.69 for Angio-iFR and 0.73 for Angio-FFR. However, investigators provided several potential reasons for this underperformance when compared to the high accuracy reported in earlier validation studies of other software. First of all, predicting resting physiology (iFR) may be more difficult for an angiography-based model than predicting hyperaemic physiology (FFR), as iFR is more sensitive to smaller frictional pressure losses that are difficult to model from a 2D image alone. Secondly, they suggest that the very high area-under-the-curve values reported for other software may be unrealistic for real-world populations because these studies were retrospective and often used highly selected, optimal angiograms, which may not reflect the performance in a more heterogeneous population. Finally, the study was conducted with a non-commercial version of the Angio-iFR/FFR software (ver 1.2) that had known technical limitations [34]. While the proposed concept holds promise, significant further software refinement is warranted before Angio-iFR/FFR can be considered as a reliable tool for clinical decision-making [34].

Despite the innovative theoretical basis of the Angio-iFR/FFR system, the performance in a real-world clinical setting was poor, as highlighted by the high rate of false positive results.

### 2.4. Single-View AccuFFRangio (sAccuFFR)

Single-view AccuFFRangio (sAccuFFR) is a variant of the AccuFFRangio system (ArteryFlow Technology, Hangzhou, China) designed to compute FFR from a single angiographic view. While the standard AccuFFRangio utilizes two projections separated by a 25° angle to reconstruct a 3D vessel model, sAccuFFR uses a single angiographic view. This approach combines single-view 3D reconstruction with CFD modelling to quantify the hemodynamic pressure drop across the stenosis. Patient-specific flow (contrast flow velocity assessed by TIMI frame count) and aortic pressure are used as boundary conditions according to the AccuFFRangio software.

The performance of sAccuFFR was evaluated in a two-centre, retrospective observational study by Wang et al. [18], comparing the performance of AccuFFRangio under different computational conditions. The patient population consisted of an initial sample of 256 patients (with 256 vessels), but 26 of them were excluded. Technical and clinical exclusion criteria were applied, and patients were excluded if the target vessels exhibited excessive foreshortening, overlap, or insufficient contrast filling, or if the lesion was located at the ostium or the left main coronary artery. Additionally, on a clinical basis, patients were excluded if they had an acute myocardial infarction (within 72 h), atrial fibrillation, or contraindications to adenosine (e.g., severe asthma); the last two clinical scenarios are known to pose challenges for wire-based FFR assessment as well.

The results demonstrated a clear trade-off between workflow simplicity and diagnostic sensitivity. While the standard two-view AccuFFRangio achieved an accuracy, sensitivity, and specificity of 93.9%, 90.7%, and 94.9%, respectively, with an area-under-the-curve of 0.971, the single-view sAccuFFR yielded a lower accuracy of 83.5% with remarkably low sensitivity (46.3%) and similar specificity (94.9%), with an area-under-the-curve of 0.870. The investigators pointed out that information losses due to the inability of the single view to capture eccentric plaque geometry or complex irregularities that are only visible from orthogonal angles led to this inconsistency between the original AccuFFRangio and single-view model.

The literature data about sAccuFFRangio are limited. Of note, the single-view approach demonstrated markedly inferior sensitivity and specificity compared to the standard two-view version.

### 2.5. X1-FFR

The X1-FFR software (SpectraWAVE Inc., USA) is commercially provided as an add-on application directly integrated into the HyperVue™ Imaging System (SpectraWAVE Inc., Bedford, MA, USA), a multimodal console that can combine intravascular imaging and physiology. The system utilizes a direct real-time angiography feed to eliminate network transfer delays, allowing for analysis in less than one minute. The use of AI algorithms reduces manual tracing for vessel segmentation. FFR calculations are performed using fluid dynamics principles to quantify the pressure drop across the interrogated stenosis based on dimensions and the estimated flow. Specific validation details are limited, but according to the FDA submission data, the diagnostic performance of X1-FFR was established in a retrospective, multi-centre clinical analysis with 285 patients and 306 vessels [35]. The lesion distribution in the coronary vessels was 65% LAD, 17% LCx, and 17% RCA. As provided by the FDA data, high concordance was demonstrated with invasive hyperaemic pressure wire measurements. The 95% limits of agreement (LoA) were narrow (−0.12, 0.12), confirming high precision across the average FFR range of 0.82. The proposed advantage of X1-FFR is procedural efficiency. The automated frame selection and elimination of DICOM transfer position the X1-FFR system as a “push-button” tool for rapid triage. However, as a single-view method, it remains susceptible to foreshortening errors if the initial angiographic acquisition angle is suboptimal, a limitation inherent to all 2D-based physiological assessments.

Unlike the published validation data of the other methods, data on the performance of the X1-FFR algorithm are not yet available in the literature. Consequently, while the internal validation data are promising and sufficient for regulatory approval, full publication of these results is awaited in order to scrutinize the patient selection criteria and performance in complex anatomical subsets (e.g., bifurcations or diffuse disease) that are often underrepresented in pre-market pivotal cohorts.

## 3. Challenges and Clinical Application

The clinical utility of any new functional assessment tool depends on its diagnostic accuracy when compared against established gold standards. A number of studies have focused on validating the performance of single-view FFR techniques, demonstrating promising results that support their integration into clinical practice. While single-view angiography-derived FFR techniques significantly simplify the physiological assessment process by reducing the need for multiple angiographic views, the quality of the chosen single angiographic projection remains a pivotal factor in diagnostic accuracy [17,21,34].

The main challenges for virtual FFR assessment (either two- or single-view) are angiographic view quality, primarily stemming from vessel overlap and foreshortening. In some projections, different coronary segments or side branches may overlap, which makes it difficult for image processing algorithms to accurately contour the target vessel lumen. Even though advanced algorithms are meant to reduce this inherent limitation, vessel overlapping can still cause inaccuracies in geometric reconstruction and flow simulations [21]. If an angiographic view significantly foreshortens the vessel, it can distort the calculated length and diameter of the stenosis, potentially leading to errors in the computational model [21]. Choosing a “least-foreshortened” or “least-overlapping” single view whenever possible can make the derived FFR value more reliable. Therefore, better input data usually gives more accurate outputs. This dependency was clearly illustrated in the sAccuFFR study, where the transition from a dual-view to a single-view model resulted in a significant drop in sensitivity (from ~90% to ~43%), underscoring that while workflow is simplified, diagnostic confidence can be compromised when the single view fails to capture complex plaque geometry [18]. For accurate lumen delineation and, more importantly, for reliable estimation of contrast flow velocity, which is a key input for the computational models [30], it is important to have good contrast opacification and a smooth, consistent contrast injection. Poor contrast quality can lead to errors in both anatomical and functional assessment. To this end, standard acquisition protocols for optimized single-view angiographic images could enhance the performance of single-view methodologies.

The true test of a functional assessment tool is not only the overall diagnostic accuracy but also the reliable performance across a range of challenging lesion subtypes, where anatomical assessment can be particularly deceptive. Single-view FFR techniques, in particular µQFR, have demonstrated promising results in these complex scenarios. Recent data have extended the clinical application of such techniques beyond stable CAD to high-risk populations, including patients with severe aortic stenosis undergoing TAVR, those requiring concomitant valvular surgery, and even in the assessment of non-culprit lesions during primary PCI for STEMI [9,10,25].

The use of single-view algorithms reduces the need for additional angiographic projection acquisitions, contrast dose, radiation exposure, and procedural time. A single angiographic projection has been anticipated to cope with the limitations of two views with potentially improved feasibility in real-world practice; in patients with tortuous anatomy, serial lesions, or diffuse disease, where it is not always possible to obtain two optimal angiographic views, single-view methods may offer a distinct advantage. Also, in the setting of ACS, e.g., STEMI patients, single-view methods may allow rapid assessment of non-culprit lesions or when planning a staged procedure guided by coronary physiology. Furthermore, these tools may help in the assessment of frail patients, such as during pre-TAVR workup, to reduce contrast volume and procedural time without the need for hyperaemic agents.

FFR-guided PCI is associated with improved clinical outcomes and reduced incidence of repeat revascularization. The virtual functional assessment, which can be achieved by integrating various imaging technologies with fluid mechanics algorithms or CFD models, is a promising alternative to the invasive FFR for guiding clinical decisions. However, most available evidence on the single-view approaches is derived from retrospective or offline analyses with selective inclusion criteria, limiting generalizability. Real-time, integrated platforms may overcome logistical barriers, but robust prospective validation is essential. Of note, in the WIFI II study [21], real-time QFR analysis failed in 5% of vessels due to poor angiographic imaging, such as severe vessel overlap or foreshortening. Conversely, performing traditional FFR measurements is technically difficult in challenging anatomies, such as a jailed side branch after main vessel stenting. In these cases, virtual FFR assessment is an alternative for these patients, and the advancement of these non-invasive techniques is crucial for enabling more widespread, patient-specific treatment decisions in interventional cardiology.

Future research must pivot from retrospective correlation studies to prospective randomized controlled trials that evaluate hard clinical endpoints (i.e., adverse cardiac events, target vessel failure) rather than just diagnostic agreement with wire-FRR. Also, the integration of these software solutions into real-time catheterization laboratory monitors—similar to the X1-FFR model—will be essential for widespread adoption and establishing them as an online decision-making tool.

## 4. Conclusions

Single-view angiography-derived FFR calculation represents a significant advancement in the physiological assessment of CAD. While all reviewed platforms share the goal of simplified physiological assessment, they diverge significantly in their operational mechanics and validation maturity. By eliminating the need for pressure wires, pharmacologic hyperaemia, and the need for multiple angiographic projections, these technologies offer a more streamlined and low-risk approach to lesion-specific ischemia evaluation. Despite these promising developments, important limitations remain. Diagnostic accuracy is highly dependent on angiographic image quality, and most validation data are derived from retrospective or selectively enrolled cohorts. Ongoing technological refinements, prospective outcome-driven trials, and real-world implementation studies will be essential to define the precise role of single-view angiography-derived FFR in contemporary practice.

## Figures and Tables

**Table 1:** Summary of single-view FFR methodologies.

Methodology	Core Principle	Validation Data (Number of Patients)	Performance	Remarks
**μQFR**	Murray’s Law (Fractal) with AI-based reconstruction	Sub-analysis of FAVOR II China (306) DIAMOND study in calcified lesions (107)	acc: 93.0% sens: 87.5% spec: 96.2% sens: 75% spec: 77.8%	Mostly retrospective analyses; available data in complex lesions (bifurcation and calcified)
**FFR2D**	Fluid mechanics (non-linear equations)	Pilot FFR2D (88)	acc: 90.9%, sens: 85.7%, spec: 93.3%	Retrospective data; semi-automatic process makes it user-dependent
**Angio-iFR/FFR**	Lumped parameter model (electric-hydraulic analogy)	REVEAL iFR (441)	sens: 77%, spec: 49%	Prospective data; high false positive rate; technical limitations noted; difficulty of modeling resting physiology from a single image for Angio-iFR
**sAccuFFR**	CFD with 3D reconstruction	Wang et al. [18] (256)	acc: 83.5%, sens: 43.3%	Retrospective data; significant drop in sensitivity compared to its 2-view counterpart
**X1-FFR**	Integrated AI & Fluid dynamics	FDA data (285)	limits of agreement: ±0.12	Lacks published data

3D: three-dimensional reconstruction, AI: artificial intelligence; acc: accuracy, CFD: computational fluid dynamics, FFR: fractional flow reserve; sens: sensitivity, and spec: specificity.

## Data Availability

This is not necessary in this case as there is no original data.
